# Substance Use–Related Admissions in Liver Transplant Recipients: A National Inpatient Sample Analysis

**DOI:** 10.1155/joot/8700177

**Published:** 2026-09-20

**Authors:** Alex Dobos Ferreira, Spencer R. Goble, Thomas M. Leventhal

**Affiliations:** ^1^ Department of Medicine, University of Minnesota, MMC 36 420 Delaware Street S.E., Minneapolis Minnesota, 55455, USA, umn.edu; ^2^ Division of Gastroenterology, Hepatology, and Nutrition, University of Minnesota, MMC 36 420 Delaware Street S.E., Minneapolis Minnesota, 55455, USA, umn.edu

**Keywords:** alcohol drinking, hospitalization, liver transplantation, mental disorders, substance-related disorders

## Abstract

**Introduction:**

While chronic complications of substance use are well‐recognized in liver transplant recipients, the ramifications of acute complications of substance use in this population are less well characterized.

**Methods:**

Using the National Inpatient Sample from 2016 to 2022, we assessed hospitalizations in liver transplant recipients for intoxication or withdrawal from any of the following: alcohol, opioids, sedatives, and stimulants. We compared these substance use–related admissions to all other liver transplant recipient admissions. Clinical outcomes were also assessed.

**Results:**

Of the 299,350 hospitalizations for liver transplant recipients, acute substance use–related complications accounted for 2375 (0.8%), with alcohol being the most common (*n* = 1455). Patients admitted for substance use–related complications were younger (mean age 52.6 vs. 60.2 years, *p* < 0.001), more likely to be white (79.1% vs. 70.6%, *p* < 0.001), and had increased rates of homelessness (2.1% vs. 0.2%, *p* < 0.001), major depressive disorder (37.7% vs. 16.7%, *p* < 0.001), and generalized anxiety disorder (37.3% vs. 13.6%, *p* < 0.001). ICU‐level care was required in 44 per 1000 substance use–related admissions, while 141 per 1000 such admissions required skilled nursing facility placement. Mean length of stay was 5.2 days, and mean cost of hospitalization was $9637.

**Conclusion:**

Liver transplant recipients admitted for substance use–related issues tend to have high rates of mental health conditions and other signals of social vulnerability. Critical illness appears uncommon in these admissions, although there is significant utilization with admission stays averaging over five days and nearly $10,000.

## 1. Introduction

With over 10,000 adult patients undergoing liver transplantation (LT) in the United States in 2023, LT is a well‐established lifesaving treatment for individuals with end‐stage liver disease and selected patients with acute liver failure when medical management has failed [1–3]. Currently, 5‐year posttransplant patient survival rates range from 79.3% to 82.2% depending on patient age and clinical factors [1]. To optimize outcomes, thorough pretransplant evaluations are necessary: medical comorbidity assessments, cardiopulmonary testing, and rigorous psychosocial evaluations to address barriers, substance use, and mental health conditions [4].

It is incredibly important to explore effective strategies to support patients who experience challenges related to substance use in the peri‐ and post‐LT period. Alcohol‐related liver disease is a well‐known disease in the chronic liver disease population and a common indication for LT [1]. There is also a high prevalence of chronic pain in liver disease and in the pre‐, peri‐, and post‐LT setting, leading to opioid prescriptions and use in these individuals, though there remain few studies on patterns of other substance use in this population [5–7]. While LT offers life‐saving physical benefits, optimizing posttransplant outcomes requires comprehensive support addressing all aspects of care, especially psychosocial needs.

Recurrent alcohol use has been studied and shown to have a negative effect on graft function, patient survival, and quality of life [8–10]. Similarly, in the evaluations of opioid use after LT, it is associated with increased risk of graft failure and increased mortality [8]. Multiple studies have shown that recurrent substance use and mental health disorders are associated with poor adherence to therapy, which carries a risk for graft rejection [7, 11–13]. However, little is known about the burden and outcomes of acute complications of substance use in this population, such as intoxication and withdrawal from alcohol and other substances. We aim to describe utilization and outcomes of substance‐related admissions in patients post‐LT.

## 2. Methods

### 2.1. Study Design and Database Description

This is a cross‐sectional retrospective study of hospitalizations for substance use–related primary diagnoses in patients with a history of LT from the years 2016 to 2022. Substance use–related primary diagnoses were defined as the presence of an ICD‐10 code for either intoxication or withdrawal from any of the following substances: alcohol, opioids, sedatives, and stimulants (Table S1). The National Inpatient Sample (NIS) was used to assess the hospitalizations. The NIS is the largest publicly available all‐payer inpatient database in the United States (https://hcup-us.ahrq.gov/nisoverview.jsp). The NIS includes information on approximately 20% of all hospitalizations in the United States which are selected via a systematic stratified sampling design. When standardized weighting procedures are applied to the NIS data, the results are intended to approximate the entire United States inpatient population. Survey‐weighted analyses were performed using the HCUP discharge weights while accounting for the NIS‐stratified cluster sampling design. LT recipients were analyzed as a survey subpopulation to obtain nationally representative estimates for hospitalizations within this subgroup. The NIS includes basic demographic information for patients along with a single primary diagnosis and up to 39 secondary diagnoses. The primary diagnosis is considered the issue to be chiefly responsible for the admission, while secondary diagnoses can be chronic conditions, acute concurrent conditions, or complications of the primary diagnosis. There is no indication of whether secondary diagnoses were present on admission in the NIS. The NIS is publicly available, so all information is deidentified and institutional review board approval was waived.

### 2.2. Study Sample and Objectives

Hospitalizations for patients 18 years of age or older with a documented history of a LT, defined by the ICD‐10 code Z94.4, were included. LT recipients admitted for nonsubstance use–related diagnoses were included to allow for comparison. Clinical and demographic characteristics of transplant recipients admitted for substance use–related primary diagnoses were compared to LT recipients admitted for other reasons. In addition, clinical outcomes were assessed including mortality, intensive care unit (ICU) level care, discharge to skilled nursing facilities, against medical advice (AMA) discharges, length of stay (LOS), and cost. ICU‐level care was defined as documented mechanical ventilation, cardiopulmonary resuscitation, vasopressor use, central line placement, or noninvasive positive pressure for greater than 24 consecutive hours.

### 2.3. Statistical Analysis

The Student’s t‐test was used to compare continuous variables, and chi‐square was used to compare categorical variables. The following clinical outcomes were reported as rates per 1000 admissions: mortality, ICU‐level care, discharge to skilled nursing facility, and AMA discharge. LOS and cost were reported as means. For each assessed outcome, corresponding 95% confidence intervals were calculated using Taylor series linearization to account for the complex survey design of the NIS. Missing data were infrequent (< 4% for all variables) (Table S2). No imputation was performed, and analyses were conducted using available observations. All statistical computations were performed using STATA, Version 17.0.

## 3. Results

### 3.1. Clinical and Sociodemographic Characteristics

A total of 299,350 hospitalizations for patients with a history of LT were identified, 2375 (0.8%) of which had a substance use–related primary diagnosis. The mean age for all admissions was 60.1 years, and 39.5% of patients were female, with the majority of patients (70.6%) being white. Patients admitted for substance use–related issues had a lower burden of comorbid disease, with a mean Charlson Comorbidity Index of 2.7 compared to 4.0 in patients admitted for other reasons (*p* < 0.001) (Table 1). Each assessed mental health diagnosis was increased in patients admitted for substance use–related issues, except for attention deficit hyperactivity disorder.

**TABLE 1:** Characteristics of substance use–related hospitalizations for patients with a history of liver transplantation.

Variable	Substance use–related admission	Nonsubstance use–related admission	*p* value
Sample size	2375	296,975	
Mean age, years (SD)	52.6 (13.2)	60.2 (13.5)	< 0.001^∗∗^
Female, %	29.5	39.6	< 0.001^∗∗^
Race, %			0.006^∗^
White	79.1	70.6	
Black	5.1	8.8	
Hispanic	10.8	14.2	
Asian	2.0	2.7	
Other	3.1	3.7	
United States region, %			< 0.001^∗∗^
Northeast	28.6	18.7	
Midwest	24.4	22.5	
South	30.5	39.2	
West	16.4	19.6	
Mean Charlson Comorbidity Index (SD)	2.7 (1.8)	4.0 (2.2)	< 0.001^∗∗^
Human immunodeficiency virus, %	1.7	0.6	< 0.001^∗∗^
Hepatitis C virus, %	12.0	8.4	< 0.001^∗∗^
Hepatitis B virus, %	0.8	1.9	0.086
Autoimmune liver disease, %	0.2	2.8	< 0.001^∗∗^
Tobacco use, %	34.7	8.8	< 0.001^∗∗^
Homelessness, %	2.1	0.2	< 0.001^∗∗^
Schizophrenia, %	1.7	0.7	0.013^∗^
Bipolar disorder, %	4.8	1.7	< 0.001^∗∗^
Major depressive disorder, %	37.7	16.7	< 0.001^∗∗^
Generalized anxiety disorder, %	37.3	13.6	< 0.001^∗∗^
Posttraumatic stress disorder, %	4.2	1.1	< 0.001^∗∗^
Attention deficit hyperactivity disorder, %	0.4	0.4	0.995
Personality disorder, %	1.3	0.2	< 0.001^∗∗^
Dementia, %	8.2	4.6	< 0.001^∗∗^
Documented medication noncompliance, %	11.6	3.8	< 0.001^∗∗^
Substance responsible for admission, %			
Alcohol	61.3	—	—
Opioid	22.7	—	—
Sedative	11.2	—	—
Stimulant	4.8	—	—

*Note:* No hospitalizations were excluded because of missing demographic data. Age was missing for 30 hospitalizations, sex for 60 hospitalizations, and race for 9245 hospitalizations. Percentages for each variable were calculated using the number of hospitalizations with nonmissing data for that variable as the denominator.

^∗^ *p* < 0.05.

^∗∗^ *p* < 0.001.

Of the assessed substances, alcohol was the responsible agent for the majority of admissions (*n* = 1455). Alcohol withdrawal accounted for 960 admissions (66.0% of alcohol‐related admissions), while alcohol intoxication accounted for 495 admissions (34.0%). Opioids were responsible for the next most admissions with 540. Of those 540, 405 (75.0%) were for intoxication, while 135 (25.0%) were for withdrawal. Sedatives were responsible for 265 admissions, while stimulants accounted for 115 admissions.

### 3.2. Clinical and Healthcare Utilization Outcomes

A total of 10 in‐hospital deaths were recorded for patients with a history of LT who were admitted for substance use–related diagnoses (overall mortality 4:1000 admissions). Of the 2375 substance use–related admissions, 105 (44:1000 admissions) resulted in the need for ICU‐level care, with rates ranging from 17:1000 alcohol‐related admissions to 87:1000 stimulant‐related admissions (Table 2). Mortality in cases that required ICU‐level care was 48:1000 hospitalizations.

**TABLE 2:** Clinical and healthcare utilization outcomes for substance use–related admissions in liver transplant recipients.

Outcome	Substance responsible for admission	Number per 1000 admissions	95% CI (per 1000 admissions)
ICU‐level care^a^	All	44	29–67
	Alcohol	17	7–41
	Opioid	93	51–164
	Sedative	75	29–185
	Stimulant	87	22–289
	Nonsubstance use–related	63	61–66

Discharge to a skilled nursing facility	Any	131	103–164
	Alcohol	110	79–151
	Opioid	139	85–218
	Sedative	245	148–379
	Stimulant	87	22–289
	Nonsubstance use–related	123	119–126

Against medical advice discharge	Any	72	51–100
	Alcohol	62	38–98
	Opioid	83	44–152
	Sedative	75	29–185
	Stimulant	130	43–336
	Nonsubstance use–related	12	11–13

**Utilization outcomes**	**Substance responsible for admission**	**Mean**	**95% CI**

Mean length of stay (days)	All	5.2	4.6–5.7
	Alcohol	5.5	4.8–6.3
	Opioid	4.6	3.6–5.6
	Sedative	4.8	3.7–5.8
	Stimulant	4.8	3.2–6.4
	Nonsubstance use–related	5.9	5.8–5.9

Mean cost (US $)	All	$9637	$8711–$10,563
	Alcohol	$9125	$7877–$10,373
	Opioid	$9943	$8278–$11,608
	Sedative	$10,705	$8476–$12,935
	Stimulant	$12,152	$7539–$16,766
	Nonsubstance use–related	$18,432	$18,057–$18,807

^a^Defined as documented mechanical ventilation, cardiopulmonary resuscitation, vasopressor use, central line placement, or noninvasive positive pressure for greater than 24 consecutive hours.

Discharge to a skilled nursing facility was recorded for 310 of the 2365 substance use–related hospitalizations that had disposition data available (131:1000). A total of 1815 (76.7%) patients were discharged to home, with the remaining discharges either being transfers to other hospitals (*n* = 70) or AMA discharges (*n* = 170). Patients admitted for sedative‐related diagnoses had the highest rate of skilled‐nursing facility discharge (245:1000 hospitalizations). Stimulant‐related admissions had the highest rate of AMA discharges (130:1000).

Mean LOS for all substance use–related hospitalizations was 5.2 days. LOS was greater than 10 days in 245 (10.3%) hospitalizations and was two days or less in 770 (32.4%) hospitalizations. Alcohol‐related hospitalizations tended to be the longest (mean LOS 5.5 days). The mean cost for all substance use–related hospitalizations was $9637. Cost was greater than $25,000 in 185 (7.8%) hospitalizations and less than $5000 in 810 (34.1%) hospitalizations. Cost was highest in stimulant‐related admissions (mean cost $12,152).

## 4. Discussion

This study identified increased rates of homelessness and mental health diagnoses in post‐LT patients admitted for substance use–related diagnoses. Although mortality and ICU‐level care were relatively uncommon in these admissions, significant health care utilization was noted, with a mean LOS greater than 5 days and 14% of patients requiring placement to a skilled nursing facility at discharge.

Prior studies have found that post‐LT substance use has not only been linked to immunosuppressive medication nonadherence with subsequent graft loss but may also contribute to missed clinic appointments and decreased social support [12, 14]. Psychiatric comorbidities and younger age have been associated with alcohol relapse in the setting of LT, and in this study, high rates of psychiatric comorbidities and a relatively young mean age were both noted in substance use–related admissions [15–18]. The use of other substances post‐LT has been relatively poorly characterized; however, in limited assessments, opioid use has been associated with psychiatric comorbidities and pre‐LT opioid use, while stimulant use disorders are frequently co‐occurring with mental health conditions [7, 19–21]. Younger age may reflect different psychosocial stressors, mental health concerns, or patterns of substance use compared to older transplant recipients, which serves as an explanation for the increased substance use–related admissions in this population.

Homelessness was documented in 2.1% of substance use–related admissions compared to just 0.2% in all other admissions. Studies have shown that patients experiencing homelessness have high rates of comorbid chronic medical illness, substance use disorder, and psychiatric comorbidities, which have also been seen in patients with cirrhosis experiencing homelessness [22–25]. Although there is extensive workup prior to LT with comprehensive substance use and psychosocial assessments, substance use disorders and psychiatric comorbidities are dynamic, chronic processes [26]. Studies that have performed an integrated approach with psychiatric resources have shown lower mortality and alcohol relapse rates post‐LT and lower relapse rates in chronic liver disease [13, 27–29]. The integration of addiction and psychiatric medicine specialists and routine screening for housing instability may help mitigate preventable hospitalizations and improve long‐term outcomes by decreasing post‐LT substance use.

Limitations of our study include reliance on ICD‐10 codes, which can lead to misclassification of diagnoses, a lack of data on timing of LT, and a lack of data on graft function. The NIS does not include medication data, and we therefore cannot determine which hospitalizations may have been related to prescribed medications. In addition, we defined substance use‐related admissions solely as admissions with a primary diagnosis related to substance use. This likely underestimates the burden of substance use‐related admissions in this population. For example, if a patient was admitted with a primary diagnosis of a mechanical fall but was also noted to be in alcohol withdrawal, they would not have been included in our study group. In addition, patients may have had multiple substance‐related issues present on admission. A balancing strength, however, is that this method ensures that we are truly capturing patients who are presenting for a direct acute complication of substance use, while including secondary diagnoses in our definition would risk inclusion of patients with resolved or chronic substance use diagnoses and would ultimately harm the reliability of our study. It should also be noted that the NIS characterizes hospitalizations, not individual patients. Numerous patients likely accounted for multiple hospitalizations, and our study does not include every LT recipient.

Overall, our study found that substance use–related admissions post‐LT are closely intertwined with mental health diagnoses, younger age, and homelessness. Although significant morbidity and mortality were rare, healthcare utilization remained substantial in substance use–related admissions in post‐LT patients, likely reflecting prolonged hospitalizations and complex care needs.

**Nomenclature**
AMA: Against medical advice
CI: Confidence interval
HCV: Hepatitis C virus
HIV: Human immunodeficiency virus
ICD‐10: International Classification of Diseases, 10th Revision
ICU: Intensive care unit
LOS: Length of stay
LT: Liver transplantation
NIS: National Inpatient Sample
OR: Odds ratio

## Funding

This research did not receive any funding.

## Conflicts of Interest

The authors declare no conflicts of interest.

## Supporting Information

Additional supporting information can be found online in the Supporting Information section.

## Supporting information


**Supporting Information** Table S1. ICD‐10 codes used to assess substance use–related hospitalizations for patients with a history of LT. Table S2. Number of missing variables for assessed patient factors and outcomes in liver transplant recipient hospitalizations.

## Data Availability

The data that support the findings of this study are available in the National Inpatient Sample at https://hcup-us.ahrq.gov/nisoverview.jsp. These data were derived from the following resources available in the public domain: National Inpatient Sample, https://hcup-us.ahrq.gov/nisoverview.jsp.
