# Supplementary material for: Substance Use–Related Admissions in Liver Transplant Recipients: A National Inpatient Sample Analysis
Source: J Transplant. 2026 Sep 20;2026:8700177. doi: 10.1155/joot/8700177 (PMC13591122; doi:10.1155/joot/8700177)
Supplement: Supplementary file 1 [file JOOT-2026-8700177-s001.docx]

**Substance Use-Related Admissions in Liver Transplant Recipients: A National Inpatient Sample Analysis**

Authors: Alex Dobos Ferreira MD, Spencer R Goble MD, Thomas M Leventhal MD

Table of Contents:

Supplementary methods: ………………………………………………………………………….	2

Table S1. ICD-10 codes used to assess substance use-related hospitalizations for patients with a history of liver transplantation

| Attention deficit hyperactivity disorder | F90 |
| --- | --- |
| Alcohol intoxication | F1015*, F1018*, F1019*, F1022*, F1025*, F1026*, F1027*, F1028*, F1029*, F1092*, F1094*, F1095*, F1096*, F1097*, F1098*, F1099* |
| Alcohol withdrawal | F1023*, F10103*, F1093* |
| Anxiety disorder | F41* |
| Autoimmune liver disease | K75.4, K74.3, K74.5, K83.0 |
| Bipolar disorder | F31* |
| Central line placement | 06HN33Z, 06HM33Z, 05H633Z, 05H533Z, 05HN33Z, 05HM33Z, 05HM3DZ |
| Continuous positive pressure ventilation | 5A094*, 5A095* |
| Dementia | F00*, F01*, F02*, F03*, F04*, G30*, G31*, R41, I6991 |
| HBV | B16.2, B16.9, B18.1, B19.1, B19.10. B19.11 |
| HCV | B17.10, B17.11, B181.2, B19.20, B19.21, Z22.52 |
| Hepatic encephalopathy | K72.91, K72.01, K72.11,  K72.90 |
| HIV | B20.0, B20.1, B20.2, B20.3, B20.4, B20.5, B20.6, B20.7, B20.8, B20.9, B21.0. B21.1, B21.2, B21.3, B21.7, B21.8, B21.9, B22.0, B22.1, B22.2, B22.7, B23.0, B23.1, B23.2, B23.8, B20, B24, Z21 |
| Homelessness | Z59.0 |
| Major depressive disorder | F32*, F33* |
| Liver transplant history | Z94.4 |
| Mechanical ventilation | 0BH17EZ, 0BH18EZ, 5A1935Z, 5A1945Z, 5A1955Z |
| Medication non-compliance | Z9112*, Z9113*, Z9114*, Z9119*, Z91A1*, Z91A2*, Z91A3*, Z91A4* |
| Opioid intoxication | F1110*, F1112*, F1114*, F1115*, F1118*, F1119*, F1120*, F1122*, F1124*, F1125*, F1128*, F1190*, F1192*, F1194*, F1195*, F1198*, F1199*, T400*, T401*, T402*, T403*, T404* |
| Opioid withdrawal | F1113*, F11123*, F1193* |
| Personality disorders | F60 |
| Post-traumatic stress disorder | F431* |
| Schizophrenia | F200, F201, F202, F203, F205, F2081, F2089, F209, F22, F23, F250, F251, F258, F259, F28, F29 |
| Sedative use | F13*, T424*, T423*, T426*, T427* |
| Stimulant use | F15*, F14*, T4368 |
| Tobacco use | F17* |
| Vasopressor use | 3E030XZ, 3E033XZ, 3E040XZ, 3E043XZ, 3E050XZ, 3E053XZ, 3E060XZ, 3E063XZ |

Table S2. Number of missing variables for assessed patient factors and outcomes in liver transplant recipient hospitalizations

| Variable | Substance use related admissions | Non-substance use related admissions | All admissions |
| --- | --- | --- | --- |
| Age, n (%) | 0 (0.0) | 30 (0.0) | 30 (0.0) |
| Sex, n (%) | 0 (0.0) | 60 (0.0) | 60 (0.0) |
| Race, n (%) | 105 (4.4) | 9,140 (3.1) | 9,245 (3.1) |
| United States Region, n (%) | 0 (0.0) | 0 (0.0) | 0 (0.0) |
| Discharge disposition, n (%) | 10 (0.4) | 11,135 (3.7) | 11,145 (3.7) |
| Length of stay, n (%) | 0 (0.0) | 5 (0.0) | 5 (0.0) |
| Cost, n (%) | 25 (1.1) | 2,755 (0.9) | 2,780 (0.9) |
